# A Retrospect

**Published:** 1912-12-28

**Authors:** 


### NATIONAL INSURANCE ACT DEPARTMENT.

### XXXVII.—A Retrospect.

The year 1912, now so fast drawing to a close, will long be remembered as the year in which the National Insurance Act came into operation. It has been a year full of many great and important events, but few of these will have such far-reaching effects as those of "The Act." The Act was presented to the nation as a completed piece of legislation as a Christmas box in the closing days of last year. In its passage through the House it met with a mixed reception. Upon its introduction its general principles were cordially approved by all parties, and the Chancellor of the Exchequer expressed the hope that the measure would not be treated on party lines, but that all would co-operate to produce a really workable system.

If the Chancellor had only acted on this principle, and allowed extensive examination and discussion, and, if necessary, the amendment of the original scheme, much of the friction that has since occurred would have been avoided. With debate stifled by the operation of the guillotine, many important sections were never discussed at all, and duly tabled amendments were never reached.

In the circumstances, the details of the measure were but faintly understood by the House as a whole, and all points of intricacy were left to be subsequently settled by Order of the Insurance Commissioners.

Thus it happened that a measure of first-rate importance, affecting directly as beneficiaries at least one-third of the entire population of the United Kingdom, and indirectly, either as employers or as taxpayers, or as both, practically the whole of the balance of the population, was placed on the Statute Book in an imperfect condition.

All matters of difficulty on which the Chancellor of the Exchequer apprehended opposition, or that might have been anticipated as likely to give rise to differences of opinion, were relegated to the Insurance Commissioners for decision.

### Ill-considered Legislation.

There were, of course, political and opportunist reasons for this haste in legislation. In our view, there was only one ground on which the haste could be justified—namely, that immediately the Bill was promised a serious check was placed upon the operations of the existing Friendly Societies, many of which found it almost impossible to secure new members, while in other cases membership actually declined. When the Bill was introduced practically all fresh transactions with the societies ceased, and thus, if the measure had not actually been carried into law, it would have left the Friendly Societies in an infinitely worse position than they had been previously, although its object was to extend the Friendly Society principle.

The Chancellor of the Exchequer had at his disposal in framing the measure the services of experienced Friendly Society officials, and of the most expert actuaries of the day. He recently informed the medical profession that, contrary to general public opinion, he had also formally consulted three deputations of medical men before the introduction of the Act. Its success, as far as it goes, is largely attributable to the closeness of the adherence of the measure to the tried experience of the great Friendly Societies, and its failure to command public esteem is to some extent due to the same cause. As the societies had only been able to obtain the support of a comparatively small section of the industrial population, it required more than a legislative enactment to render compulsory insurance under a similar system truly popular among the masses.

### Uncertainty as to Commencement.

The opposition to the Act did not die down with its entry on the Statute Book, and throughout the early months of the year considerable uneasiness was caused by the possibility of its repeal. This possibility was perhaps a remote one, but nevertheless it led to hesitation on the part of many who would otherwise have taken early steps in completing arrangements for the administration of the Act.

The commencement of the operation of the Act was another matter of uncertainty, which did not facilitate the conduct of efficient administration. By special provisions in the Act its commencement might have been any date prescribed by Order of His Majesty in Council from July 15, 1912, to January 1, 1913. It was only when, so late as June, the Government held firmly to their decision that the Act should come into operation at the earliest possible moment that it was generally realised that the Act had come to stay.

In the months of May and June the great Friendly Societies were the loudest in their complaints as to the impossibility of setting up effective machinery in time, but by stress of circumstances work has been accomplished that at one time seemed impossible.

### The Formation of Approved Societies.

The first operation in regard to the administration of the Act was the formation of approved societies. To facilitate the process several sets of model rules were drawn up by the Insurance Commissioners, and these were largely adopted with such modifications as were necessary by the majority of societies.

The first list of societies that had been approved by the Insurance Commissioners was issued at the beginning of the month of June, and then it was that the struggle for business really commenced. Official lecturers appointed by the Insurance Commissioners had been delivering lectures on the Act at important centres up and down the country; their efforts had been backed up by official leaflets distributed by the million, and the press generally had taken an active share in elucidating its mysteries. It was not, however, until the larger approved societies got to work and began the personal canvass for members that the Act became a living reality to the population in general.

It was then that they began to realise how great a change had been effected by a single piece of social legislation, and their interest having been aroused, it became more or less an easy matter to persuade them that their interests were best served by joining an approved society rather than becoming deposit contributors.

We took our share in the effort to obtain for our readers the very best that was possible to be obtained for them under the Act, and our columns were opened to correspondents, to whom answers of an expert were furnished. In particular, it will be remembered that we advocated the Nurses' Insurance Society to those of our readers who belong to the nursing profession, and it is gratifying to learn that that Society has secured a large and representative membership throughout the country.

The work involved in securing members for the approved societies was enormous, and the amount of detail that has had to be mastered in dealing with the registration of membership and the crediting of contributions to the proper membership accounts is not even now realised, except by those who have had to deal with the matter in actual practice.

### The Work of the Insurance Committees.

Not only did the Act require the formation of approved societies, but it necessitated the setting up of an Insurance Committee for each County and County Borough. These Committees were brought into being at the end of June, and their operations have commenced in the administration of sanatorium benefit. Within the past few weeks they have taken the first steps towards the formation of the panels of doctors who are prepared to take service under the Act for the administration of medical benefit.

This action on the part of the Insurance Committees was unduly belated, on account of the protraction of the negotiations between the Chancellor of the Exchequer and the medical profession. These negotiations have engaged public attention so much during the past few weeks that it seems almost unnecessary to say anything further in reference thereto. It may, however, be recalled that representations were made by the profession against the terms for medical service embodied in the Bill, and on the strength of those representations considerable modifications were made in the original plan. In regard to remuneration for services rendered, for which there is no direct provision in the Act, the final offer of the Government goes more than half-way to meeting the demand of the British Medical Association, and in so doing it is estimated that an additional sum of £1,650,000 a year will have to be provided by the State out of general taxation.

### The Work of the Insurance Commissioners.

The appointments on the various Insurance Commissions have not been sinecures. A vast machinery of a very complicated character had to be brought into being and set in motion at very short notice. Taking all things into consideration, the work that has been accomplished has been thoroughly and efficiently done. The duties of the Commissioners have been of a very complex character. On the one hand, they involved the necessity of regulating the expenditure of public money, and, on the other hand, they were concerned in giving to all insured persons the full benefits of the Act. Beyond these duties the Commissioners were invested with powers to regulate the operations of the approved societies and Insurance Committees. Furthermore, all the detail in regard to the method of collecting contributions had to be elaborated. The result is seen in the insurance cards and books now in the hands of all insured persons. The Commissioners have had to draw up tables of reserve rules, and of contributions for voluntary contributors. Benefit schemes for aliens and for those over sixty-five years of age at the commencement of the Act have had to be drawn up, and special orders have been framed to meet the cases of outworkers and those engaged in the mercantile marine. Many problems of an intricate character relating to the application of the Act to individuals have had to be solved, and by no means the least of their multifarious duties has been that of holding an even hand between the jealousies of rival societies.

### The Failure of Organised Opposition.

It is remarkable that opposition to the Act on the part of employers and insured persons that had been so freely predicted, and which led to the formation of resistance leagues, has been so insignificant and ineffective. A few prosecutions under the penal clauses of the Act soon showed that the Government intended the law to be strictly enforced, and it has become apparent that resistance is impossible, and that those who feel aggrieved must proceed by constitutional methods to obtain such amendments in the Act as may prove to be necessary as a result of its practical operation.

### The Commencement of Benefits.

The Act is emerging from its most unpleasant phase—that in which contributions have been payable without the corresponding right to benefit. While the future cannot yet be anticipated without misgiving, especially in regard to the administration of medical benefit, and the effect that is foreshadowed in the matter of treatment of insured persons in voluntary hospitals, we are confident that before very long the principles of National Insurance will be vindicated. It is no mean achievement to have provided the way whereby, in his hour of need, arising through sickness and incapacity for work, a workman is able to look forward with certainty to an allowance of 7s. 6d. or 10s. a week. Small though the sum may be, it represents a great deal when there is no other source of income.

---

**NOTE.—Questions and Correspondence on all doubtful points are invited, and should be addressed to the Editor, The Hospital, 28, 29 Southampton Street, Strand, London, W.C., and marked "Insurance."**

